# Efficacy of digital cognitive behavioural therapy for symptoms of generalised anxiety disorder: a study protocol for a randomised controlled trial

**DOI:** 10.1186/s13063-020-4230-6

**Published:** 2020-04-23

**Authors:** J. Gu, C. B. Miller, A. L. Henry, C. A. Espie, M. L. Davis, R. Stott, R. Emsley, J. A. J. Smits, M. Craske, K. E. A. Saunders, G. Goodwin, J. R. Carl

**Affiliations:** 1 Big Health Inc., San Francisco, CA, USA, London, UK; 2 Department of Psychiatry, University of Oxford, Oxford, UK; 3 Sleep and Circadian Neuroscience Institute, Nuffield Department of Clinical Neurosciences, University of Oxford, Oxford, UK; 4 King’s College London, Department of Biostatistics and Health Informatics, Institute of Psychiatry, Psychology, & Neuroscience, London, UK; 5 Department of Psychology, The University of Texas at Austin, Austin, TX USA; 6 Anxiety and Depression Research Centre (ADRC), University of California, Los Angeles, CA USA

**Keywords:** Randomised controlled trial, RCT, Digital, Smartphone, Cognitive behavioural therapy, CBT, Generalised anxiety disorder, GAD, Anxiety

## Abstract

**Background:**

Generalised anxiety disorder (GAD) is a chronic and disabling condition with considerable personal and economic impact. Cognitive behavioural therapy (CBT) is a recommended psychological therapy for GAD; however, there are substantial barriers to accessing treatment. Digital CBT, in particular smartphone-delivered CBT, has the potential to improve accessibility and increase dissemination of CBT. Despite the emerging evidence of smartphone-based psychological interventions for reducing anxiety, effect size scores are typically smaller than in-person interventions, and there is a lack of research assessing the efficacy of smartphone-delivered digital interventions specifically for GAD.

**Methods:**

In the DeLTA trial (DigitaL Therapy for Anxiety), we plan to conduct a parallel-group superiority randomised controlled trial examining the efficacy of a novel smartphone-based digital CBT intervention for GAD compared to a waitlist control. We aim to recruit 242 adults (aged 18 years or above) with moderate-to-severe symptoms of GAD. This trial will be conducted entirely online and will involve assessments at baseline (week 0; immediately preceding randomisation), mid-intervention (week 3), post-intervention (week 6; primary end point) and follow-up (week 10). The primary objective is to evaluate the efficacy of the intervention on GAD symptom severity compared to a waitlist control at post-intervention. Secondary objectives are to examine between-group effects on GAD at follow-up, and to examine the following secondary outcomes at both post-intervention and follow-up: 1) worry; 2) depressive symptoms; 3) wellbeing; 4) quality of life; and 5) sleep difficulty.

**Discussion:**

This trial will report findings on the initial efficacy of a novel digital CBT intervention for GAD. Results have the potential to contribute towards the evidence base for digital CBT for GAD and increase the dissemination of CBT.

**Trial registration:**

ISRCTN, ISRCTN12765810. Registered on 11 January 2019.

## Background

Generalised anxiety disorder (GAD) is a chronic and disabling condition with a lifetime prevalence of 5–8% [1–3] and most patients continue to experience symptoms after 6 to 12 years [4]. GAD is characterised by excessive and persistent anxiety and worry that is difficult to control [5]. In addition to the considerable impact it has on the lives of individuals, GAD has serious economic consequences. It is associated with increased use of health care services, medical costs, absenteeism from work and decreased work productivity [6–8].

Cognitive behavioural therapy (CBT) is a recommended first-line treatment for GAD [9–12]. CBT for GAD encompasses a range of cognitive and behavioural components, including cognitive restructuring, imaginal exposure, situational (in vivo) exposure, stimulus control, applied relaxation, self-monitoring and psychoeducation [13–15]. Meta-analyses of randomised controlled trials (RCTs) show that, when compared to control conditions, CBT for GAD significantly improves anxiety, with large effect size scores [16, 17]. Despite this evidence base for CBT, availability is limited due to substantial barriers to accessing this treatment. These include insufficient numbers of trained therapists, costs, waiting lists, incompatible scheduling, distance from services and stigma [18, 19]. Digital CBT, or CBT delivered primarily via software applications on digital devices (e.g. computers, tablets, and smartphones) [20], provides a solution to help overcome treatment accessibility barriers and has the potential to increase dissemination of CBT. The importance of using digital technologies for improving mental health care is now well recognised [21] and there are a number of advantages offered by digital CBT. Digital CBT is cost effective [22, 23] scalable across people and geographies, and may reduce additional difficulties with attending appointments in person such as time constraints and stigma [18, 19]. Additionally, using algorithms, digital CBT has the potential to not only provide standardised and reliable, high-quality interventions for anyone, but can also be tailored to individual needs [20] thereby offering personalised psychological therapy.

The widespread uptake and use of smartphone technologies in our daily lives has increased interest in the use of these devices to deliver psychological therapies [24, 25]. In a survey of smartphone ownership conducted in 2019, 81% of US adults were found to own a smartphone [26] and smartphones are increasingly used by people to help address mental health problems [27]. They are therefore regarded as ‘next-generation’ platforms for delivering psychological therapies [21, 25], and the evidence base for the efficacy of smartphone-based interventions is growing. A recent systematic review and meta-analysis identified nine RCTs examining the effects of psychological therapies delivered primarily via smartphone on anxiety outcomes [28]. Findings showed that, compared to waitlist control conditions, smartphone interventions significantly reduced symptoms of anxiety, with a medium effect size. A more recent meta-analysis identified 29 RCTs examining the effects of smartphone interventions on generalised anxiety symptoms and found these to be superior to any control condition, with a small effect size [29].

Given that the effect size scores for in-person CBT for GAD are typically in the large range [17], there is clearly room for improvement in the efficacy of smartphone-based digital interventions. Furthermore, only four trials from the systematic review of Firth et al. [28] evaluated smartphone-based interventions designed to target anxiety. None of these four trials assessed efficacy for improving GAD symptoms, and only two of the four interventions were based on CBT, a recommended psychological therapy for GAD [9–12]. This is an important omission to address given the considerable personal and economic impact of GAD and the aforementioned difficulties associated with accessing CBT for GAD.

The current protocol is for a parallel-group superiority RCT examining the initial efficacy of a novel digital CBT intervention for GAD compared to a waitlist control for adults with at least moderate GAD symptom severity. The digital CBT intervention, Daylight, is fully automated (i.e. standalone functioning without human input) and is delivered entirely via a smartphone application (app). This form of digital CBT takes advantage of the wide uptake of smartphone devices and offers the fewest barriers to accessibility and greatest potential for scalability [20]. A waitlist control group comparison is appropriate to test the efficacy and safety of this new intervention in this population [30].

The primary objective is to evaluate the initial efficacy of a smartphone digital CBT intervention compared to waitlist control on self-reported GAD symptom severity at post-intervention (6 weeks from randomisation). Secondary objectives are to examine the effects compared to a waitlist control on GAD symptoms at follow-up (10 weeks from randomisation), and to examine effects on the following secondary outcomes at both post-intervention and follow-up: 1) worry; 2) depressive symptoms; 3) wellbeing; 4) participant-specific quality of life; and 5) sleep difficulty.

The primary hypothesis is that smartphone digital CBT intervention will be significantly more effective than waitlist control at treating GAD symptoms at post-intervention (6 weeks from randomisation). The secondary hypotheses are: 1) digital CBT will be significantly more effective than waitlist at improving GAD symptoms at follow-up (10 weeks from randomisation); 2) digital CBT will be significantly more effective than waitlist at improving the following outcomes at post-intervention (6 weeks from randomisation): worry, depressive symptoms, wellbeing, quality of life, and sleep difficulty; and 3) digital CBT will be significantly more effective than waitlist at improving the following outcomes at follow-up (10 weeks from randomisation): worry, depressive symptoms, wellbeing, quality of life, and sleep difficulty.

## Methods

### Design

This study is a parallel-group superiority RCT which will examine the efficacy of a novel smartphone-delivered digital CBT intervention for GAD compared to waitlist control for treating symptoms of GAD in adults with moderate-severe symptom severity. The DeLTA (DigitaL Therapy for Anxiety) trial will be conducted entirely online.

### Sample size

The sample size calculation was conducted using G*Power 3 [31]. The study aims to recruit 242 participants with GAD, with 121 participants randomised to each arm. This recruitment target accounts for approximately 30% attrition, based on the range of dropout rates from previous RCTs comparing smartphone-based digital interventions for anxiety to waitlist (19% to 45%; [32–34]). The sample size was determined based on an estimated medium effect on post-intervention scores on the seven-item Generalised Anxiety Disorder questionnaire (GAD-7) (Cohen’s *d* = 0.50) between the digital CBT and waitlist arms, with 90% power, a 1:1 allocation ratio, and with *α* set at 0.05. The effect size is based on a recent meta-analysis of RCTs of smartphone-delivered psychological treatment for anxiety versus waitlist which demonstrated a small-to-moderate (Hedges’ *g* = 0.33) between-group post-intervention effect on anxiety symptoms when compared to any active or waitlist/inactive control condition [28]. When comparing smartphone interventions with only waitlist/inactive control conditions, Hedges’ *g* increased to 0.45. The effect size of 0.50 corresponds to a minimally clinically significant 2-point change on the GAD-7 and a standard deviation of 4 in this population [35].

### Participants

For inclusion in this trial, participants must: 1) be aged 18 years or older; 2) score 10 or higher on the GAD-7 [35] indicating at least moderate GAD symptom severity; 3) screen positive for a GAD diagnosis on a digital version of the Mini-International Neuropsychiatric Interview (MINI) version 7 for DSM-5 [36], followed by telephone verification (see Procedure below for details); 4) be either not on prescription medication for anxiety, depressive symptoms, or poor sleep, or on a stable dose for at least 4 weeks; and 5) must not be currently receiving or have previously received CBT for anxiety in the last 12 months (this inclusion criterion was included after a modification was made by the ethical review committee after recruitment start). We will exclude participants who self-report: 1) being diagnosed with any of the following conditions: schizophrenia, psychosis, bipolar disorder, seizure disorder, substance use disorder; 2) having recent trauma to the head or brain damage; 3) having severe cognitive impairment; 4) having serious physical health concerns necessitating surgery or with a prognosis of less than 6 months; or 5) being pregnant. Individuals with other anxiety or related disorders (e.g. panic disorder, social anxiety disorder, and so forth) were included in the study as long as they endorsed worry (consistent with a GAD diagnosis) as their primary concern assessed at telephone screening.

Participants will be recruited online from the UK and the USA. A number of recruitment strategies will be used, including online, print, and broadcast media advertisements. Participants will receive 10 GBP in Amazon.com gift vouchers (or USD equivalent at the time of payment) in exchange for completing each of the core assessments following baseline (mid-intervention, post-intervention, and follow-up), and a bonus of 10 GBP (or USD equivalent) in vouchers for completing all assessments. Therefore, each participant can receive up to a total maximum of 40 GBP (or USD equivalent) in vouchers. Payment will be made at the end of the core study period for each participant (after 10 weeks from randomisation). If participants withdraw from the study, payment will be made for completion of any assessments at the point of withdrawal. Participants may withdraw themselves from the study for any reason at any time. The study investigator may also withdraw participants from the study to protect their safety and/or if they are unwilling or unable to comply with required study procedures after consultation with the study investigator team.

### Randomisation and allocation concealment

Participants will be assigned to the digital CBT or waitlist arm by blocked randomisation with a 1:1 allocation ratio. Randomisation will be carried out and the allocation sequence generated automatically upon completion of baseline measures using the randomisation function within Qualtrics Survey Software (Qualtrics, Provo, UT, USA, [37]). Members of the research team will be unable to influence randomisation and will be concealed from future assignments.

### Blinding

All assessments will be completed by participants online, independent from members of the research team, which will reduce the risk of bias associated with researcher-administered assessments. Participants will be informed of their randomly allocated condition (digital CBT or waitlist control) and will not be blind to group allocation. The trial coordinator will not be blind to group allocation as they will monitor uptake (download) of the intervention by participants. Participants who do not download the intervention will be reminded first by a daily email reminder for up to 3 days and then contacted by telephone. All other members of the research team will be blind to allocation. Participant contact with the trial coordinator will be limited to standardised emails containing instructions for completing online assessments and accessing digital CBT and will not cover treatment content or clinical support. Data analyses will be conducted by an independent external statistician who is not involved in the management of this trial. The statistician will be blind during the study and subgroup-unblind (groups labelled as ‘A’ and ‘B’) during the statistical analysis.

### Assessment points

Online assessments for the core study period will occur at 0 (baseline), 3 (mid-intervention), 6 (post-intervention) and 10 weeks (follow-up) from randomisation. Waitlist control participants will receive access to the digital CBT app after the follow-up assessment point (after 10 weeks from randomisation). In order to understand if effects persist over time, participants randomised to the intervention arm will also be invited to complete an optional longer-term uncontrolled assessment online at 6 months from randomisation without compensation. Table 1 presents details of the self-reported measures administered at each assessment point. After randomisation, participants will have up to 2 weeks to complete each questionnaire assessment battery. The trial coordinator will make every reasonable effort to follow the participant for the entire study period (from consent to the final follow-up assessment) using both email and telephone calls. To promote outcome data completeness, where necessary participants will be reminded to complete assessments first by a daily email reminder for up to 3 days and then by telephone for each overdue assessment. Participants who do not complete a study assessment will not be withdrawn from the study and will continue to be asked to complete any subsequent assessments. All online surveys will be hosted and stored on Qualtrics. Potential errors with data entry will be minimised as data will be entered by participants online.

**Table 1:** Administered self-report measures and timeline

Measure	Screen	Study period			
		Baseline (week 0) and randomisation	Mid-intervention (week 3)	Post-intervention (week 6)	Follow-up (week 10)^**a**^
Basic screening questions (age, pregnancy)	X				
Medical and psychiatric history questions	X				
Prescription medication use	X				
MINI for GAD	X				
GAD-7	X		X	X	X
Demographic questions		X			
Credibility/expectancy questionnaire		X			
PHQ-9		X	X	X	X
PSWQ		X	X	X	X
SCI-8		X	X	X	X
WEMWBS		X		X	X
Patient-Generated Index		X		X	X
Modified symptom checklist				X	
Treatment satisfaction questions^b^				X	
Concomitant treatment		X	X	X	X

*GAD* generalised anxiety disorder, *GAD-7* seven-item Generalised Anxiety Disorder questionnaire, *MINI* Mini-International Neuropsychiatric Interview, *PHQ-9* nine-item Patient Health Questionnaire, *PSWQ* Penn State Worry Questionnaire, *SCI-8* eight-item Sleep Condition Indicator, *WEMWBS* Warwick–Edinburgh Mental Wellbeing Scale

^a^Participants randomised to the intervention arm will additionally be invited to complete a similar uncontrolled follow-up assessment online 6 months from randomisation

^b^Measure will be administered to participants in the intervention arm only

### Procedure

**Fig. 1:**
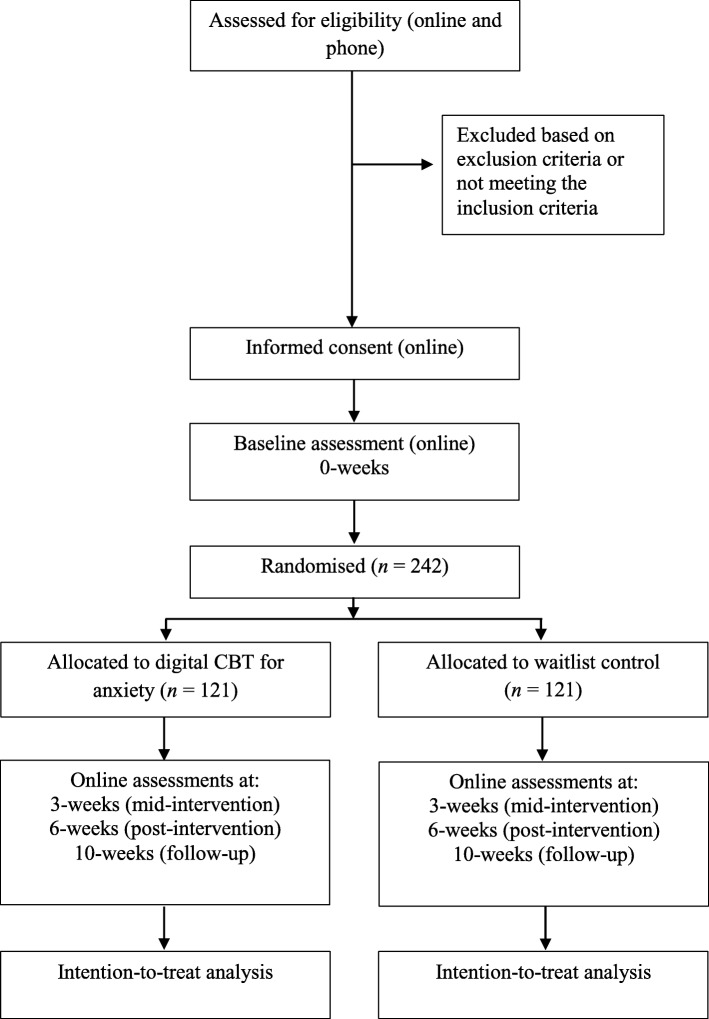
Summary of the trial design for the DigitaL Therapy for Anxiety (DeLTA) study. CBT cognitive behavioural therapy

The Consolidated Standards of Reporting Trials (CONSORT; [38]) diagram displaying participant flow through the trial is shown in Fig. 1. Participants who respond to advertisements will be directed to the online participant information sheet and asked to consent to and complete an online screening survey as a first step in assessing eligibility against inclusion and exclusion criteria. Screening questions will assess age, GAD symptom severity (GAD-7), GAD diagnosis (MINI for GAD), medical and psychiatric history, prescription medication use and pregnancy. Eligible participants who meet inclusion criteria assessed above by the online screening survey will then be contacted by a member of the research team to take part in a brief 10-min telephone call. During this call, study procedures will be explained, any questions about the study will be answered, and we will verify participant reports of GAD against criteria from the Structured Clinical Interview for DSM-5 [39], ensuring that the participant feels their GAD symptoms are their current primary area of concern. All potential participants were informed (through study advertising, the participant information sheet and during the telephone conversation) that they should aim to use the app daily throughout the study period. Eligible participants who are willing to participate are then emailed a link to the online consent form.

Participants who consent will be allocated a unique identification code to use when completing all subsequent online surveys to ensure that identifiable information provided as part of consent is not directly linked to survey responses. This unique identification code will be used for data files to ensure confidentiality. Participants will receive an email asking them to complete an online baseline survey containing a battery of self-report measures (see Table 1). Upon completion of the baseline survey, participants will be automatically randomised to either the digital CBT or waitlist control condition and informed of their allocation by automated email. All participants allocated to digital CBT will receive immediate access to the intervention and access will not be withdrawn (i.e. participants are welcome to continue using the app after conclusion of their participation in the study). All waitlist control participants will be given access to digital CBT after the final controlled follow-up assessment point (after 10 weeks from randomisation).

### Digital CBT intervention

The intervention involves interactive and tailored delivery of digital CBT for worry and anxiety via a smartphone-based app, Daylight (https://www.bighealth.com/daylight). The app provides an interactive and media-rich experience and includes supportive visuals and brief animations. Daylight is a voice-led experience, in which a virtual therapist guides the user through intervention content. Throughout the intervention, participants are asked to complete questions within the app about their anxiety and other aspects of their experience (e.g. mood, sleep). Personalisation is built in using algorithms to tailor the intervention based on responses to questions and user’s progress.

Daylight was developed in collaboration with leading experts in the area of CBT for GAD and anxiety disorders. The content is based on evidence-based CBT techniques for the treatment of GAD, including psychoeducation, stimulus control, applied relaxation, cognitive restructuring, imaginal exposures and self-monitoring of progress. The programme is designed to be self-paced and includes four modules, each lasting up to 20 min, and shorter practice versions of the same techniques (lasting approximately 5 min). Modules are accessed sequentially, with access to a subsequent module available on completion of the previous module. The app provides feedback and troubleshooting based on the user’s feedback during the exercises as well as personalised recommendations for how the techniques may be applied in the user’s life. At the start of the programme, Daylight recommends that users aim to use the app daily to practice techniques; accordingly, the modules and practice exercises can be repeatedly accessed (as per the user’s preference) for reinforcement of specific techniques. Optional notifications in the form of emails (once weekly for the first few weeks of use), text messages (twice weekly) and in-app push notifications (of varied frequency) are used to prompt use of the app. The digital CBT intervention allows for monitoring of engagement through the automatic capture of objective usage statistics including the number of modules completed, frequency of access, and length of time taken to complete all modules.

### Measures

Outcomes are self-reported and measured online at screening or baseline (week 0; immediately preceding randomisation), mid-intervention (3 weeks), post-intervention (6 weeks) and follow-up (10 weeks). Measures included are indicated below and in Table 1.

#### Primary outcome

The primary outcome will be GAD symptom severity measured using the GAD-7 [35]. The GAD-7 will be administered at all time points. Items ask participants to indicate how often they have been bothered by symptoms over the last 2 weeks. Responses are given on a four-point Likert scale ranging from 0 (“not at all”) to 3 (“nearly every day”). Items are summed and total scores range from 0 to 21. The GAD-7 has been shown to have good validity and reliability, including internal consistency, test–retest reliability and construct validity [35].

#### Secondary outcomes

In addition to scores on GAD-7, we will examine whether or not each participant demonstrated remission and reliable remission on the GAD-7 from baseline to 6 weeks post-intervention and baseline to 10 weeks follow-up. We will report and compare between groups the percentage of participants demonstrating remission (scores of <10 on the GAD-7; [35]) and reliable remission where participants experience remission and a change score reduction of ≥5 [35], which is greater than the known unreliability of the measure [40], at both time points.

#### Worry

Worry will be measured using the 16-item Penn State Worry Questionnaire (PSWQ; [41]). The PSWQ will be administered at all time points. Each item is rated using a five-point Likert scale ranging from 1 (“not at all typical of me”) to 5 (“very typical of me”). Total scores range from 16 to 80, with higher scores indicating greater degree of worry. The PSWQ has been shown to have good validity and reliability, including internal consistency, factorial validity and convergent and discriminant validity [42].

#### Depressive symptoms

Depressive symptoms will be assessed using the nine-item Patient Health Questionnaire (PHQ-9; [43]), administered at all time points. Items ask participants to indicate how often they have been bothered by symptoms over the last 2 weeks. Responses are given on a four-point Likert scale ranging from 0 (“not at all”) to 3 (“nearly every day”). Total scores range from 0 to 27, with higher scores indicating greater severity of symptoms. The PHQ-9 has been shown to have good validity and reliability, including internal consistency, test–retest reliability, sensitivity to change and criterion validity [44]. Participants experience remission if they score <10 and reliable remission if they both remit and reduce by ≥6 [45].

#### Sleep difficulty

Sleep difficulty will be measured using the eight-item Sleep Condition Indicator (SCI-8; [46]), administered at all time points. Total scores range from 0 to 32, with higher scores indicating better sleep. The SCI-8 has been shown to have good validity and reliability, including internal consistency, convergent validity and sensitivity to change [46]. Participants experience remission if they score >16 and reliable remission if they both remit and reduce by ≥7 [46].

#### Wellbeing

Positive mental wellbeing will be assessed using the 14-item Warwick–Edinburgh Mental Wellbeing Scale (WEMWBS; [47]). Items ask participants to indicate how often they have particular experiences over the last 2 weeks. Responses to items are given on a five-point Likert scale ranging from 1 (“none of the time”) to 5 (“all of the time”). Total scores range from 14 to 70, with higher total scores indicating greater wellbeing. The WEMWBS has good validity and reliability, including factorial validity, internal consistency, test–retest reliability, and convergent and discriminant validity [47].

#### Quality of life

Quality of life will be assessed using the individualised Patient-Generated Index measure [48]. The Patient-Generated Index will consist of two parts in which participants are asked to: 1) identify the three most important areas of their lives affected by their anxiety; and 2) for each area identified, indicate the degree to which this area has been affected by their anxiety in the past month, from 0 (“the worst you could imagine”) to 100 (“exactly as you would like to be”). The Patient-Generated Index has been shown to have good validity and reliability [49].

### Further measures

#### Treatment credibility and expectancy

Credibility of and expectancy of benefit from the digital CBT intervention will be assessed using the six-item Credibility/Expectancy Questionnaire [50] administered at baseline only. This questionnaire has been found to have good internal consistency and test–retest reliability [50].

#### Treatment satisfaction

Treatment satisfaction will be assessed in participants randomised to the intervention arm by the following questions administered at post-intervention: 1) “How would you rate your overall satisfaction with the programme?” (ranging from 0 (totally dissatisfied) to 10 (totally satisfied)); 2) “What could be better about this treatment programme?”; 3) “What did you like and enjoy about the treatment programme?”; 4) “In what ways did the treatment programme help you to reduce your anxiety?”; 5) “At any point during the treatment programme did you consider stopping using it? When and Why?”; and 6) “Was the content of the treatment programme specific enough to your needs?”

#### Assessment of safety

We will record the occurrence of any serious adverse events or adverse events reported to the trial coordinator at any point from study entry until the participant has left the study. A serious adverse event is defined as any untoward medical occurrence that is believed by the investigators to be causally related to digital CBT and results in any of the following: life-threatening condition (that is, immediate risk of death); or severe or permanent disability, prolonged hospitalisation, or a significant hazard as determined by the trial management committee. The study will monitor for potential adverse effects through frequent Anxiety and Depression symptom questionnaires (GAD-7 and PHQ-9). A modified symptom checklist [51] will also be used to document potential somatic and psychological side effects. The questionnaire is administered at post-intervention only and asks participants to indicate whether or not they experienced any of 14 pre-specified unwanted symptoms (e.g. low mood, feeling agitated) or other non-listed unwanted symptoms at any point during the treatment period. The original version of the symptom checklist asks participants to report on the development of any unwanted symptoms while taking part in a programme. In order to ensure that the questionnaire is also applicable to the waitlist condition (who will not have access to the programme until 10 weeks after randomisation), the questionnaire was modified after recruitment start to ask whether or not participants developed any unwanted symptoms more generally over the last 6 weeks. The questionnaire also asks to what extent any unwanted symptoms interfered with everyday functioning using a five-point Likert scale from 0 (“not at all”) to 4 (“very much”).

#### Concomitant treatment

The administration of other interventions and medications to address GAD is not prohibited after randomisation. Concomitant treatment will be assessed using the following questions administered at all time points from baseline onwards. First, “How many days over the last 3 weeks did you see a treatment provider about your anxiety” and, if yes, “what type of treatment did you pursue?”; this is captured from predefined responses (e.g. “general advice from my family physician or general practitioner or cognitive behavioural therapy, etc.”). If participants select CBT, they will then be asked to select which techniques they have used from a further predefined list. This question was included after a modification was made by the ethical review committee after recruitment start. Second, participants will also be asked “How many days in the last 3 weeks have you taken medications for anxiety that were prescribed by your doctor or not prescribed by your doctor?” (with the follow-up question “Please list these medications and the dosage”).

### Data management

The trial investigators and the trial statistician will have access to the final cleaned dataset. The project dataset will be collected and stored online in the study web portal created for the study by Qualtrics Survey Software (Qualtrics, Provo, UT, USA), and all data will be password protected. Data integrity will be enforced through a variety of mechanisms including data rules, range checks and consistency checks against data already stored in the database. All data will be treated as confidential. To ensure confidentiality, data dispersed to project team members will be blinded of any identifying participant information. Questionnaire data will be de-identified by a unique coded participant identification number only (assigned at consent into the study) to maintain confidentiality. Participant data linkage information will be stored in a single digital file separate from questionnaire research data. This file will be encrypted and stored on a secure server with limited user access and password protection for members of the study team only. Minimal personal identifiable information (e.g. name, email) will be captured and will only be made accessible (password protected) to the research study team. These data will be deleted at the completion of the study on publication or public release of the results. We will only hold contact information of participants who give optional consent to being contacted again in the future and will retain these records for 7 years after the release of this work. The final anonymised research data will be archived with the Oxford Research Archive (ORA-Data) at the University of Oxford for longer term storage (at least 7 years) after publication or public release of the results.

### Statistical analysis

Analyses will be carried out using Stata [52]. In accordance with the CONSORT statement [38], we will report all participant flow in each arm (see Fig. 1). Descriptive statistics of participant recruitment, study dropout and intervention engagement will be reported. Baseline characteristics will be presented by randomised group and summarised by means and standard deviations for continuous measures, or number and percentage for categorical measures.

To test the primary hypothesis, treatment effects on GAD symptom severity (GAD-7) will be estimated using linear mixed models fitted to data at all core time points (baseline, 3 weeks mid-intervention, 6 weeks post-intervention, 10 weeks follow-up). Fixed effects will be baseline assessment for GAD-7, randomised group, time (categorical), and time by group interactions. Participant ID will be included as random intercepts to allow for repeated measures. Marginal treatment effects will be estimated for GAD-7 at each time point and reported separately as mean-adjusted differences in scores between the randomised treatment groups with 95% confidence intervals and two-sided *P* values. The analysis will use statistical techniques for handling missing data under a missing-at-random assumption. Secondary hypotheses will be tested using analogous analyses. For the binary secondary outcome (whether participants achieved remission and reliable remission on GAD-7, PHQ-9 and SCI-8 outcomes), the same approach will be followed using logistic mixed models. All primary and secondary analyses will be conducted on an intention-to-treat basis, including all participants who were randomised, with no planned interim analysis.

Additional sensitivity analyses will be conducted to test primary and secondary hypotheses using data from participants who receive and engage with the intervention, defined as those who: 1) download the app; 2) complete at least two modules of digital CBT; and 3) complete all modules. Separate sets of analyses will be conducted for each of these three categories of intervention completion.

Exploratory analyses will examine mechanisms underlying the effect of digital CBT compared to waitlist control on GAD symptom severity using causal mediation methods based on parametric regression models. This will examine whether changes in worry (PSWQ), depressive symptoms (PHQ-9) and sleep difficulty (SCI-8) during the intervention period (from baseline to 3 weeks mid-intervention) significantly mediate the effect of digital CBT compared to waitlist on changes in GAD symptom severity (GAD-7) from baseline to 6 weeks post-intervention. Exploratory moderation analyses will also be conducted to examine whether the between-group effect on GAD symptom severity is moderated by baseline variables (e.g. demographic variables, baseline levels of anxiety).

All analyses will be pre-specified in a detailed statistical analysis plan.

### Dissemination

**Fig. 2:**
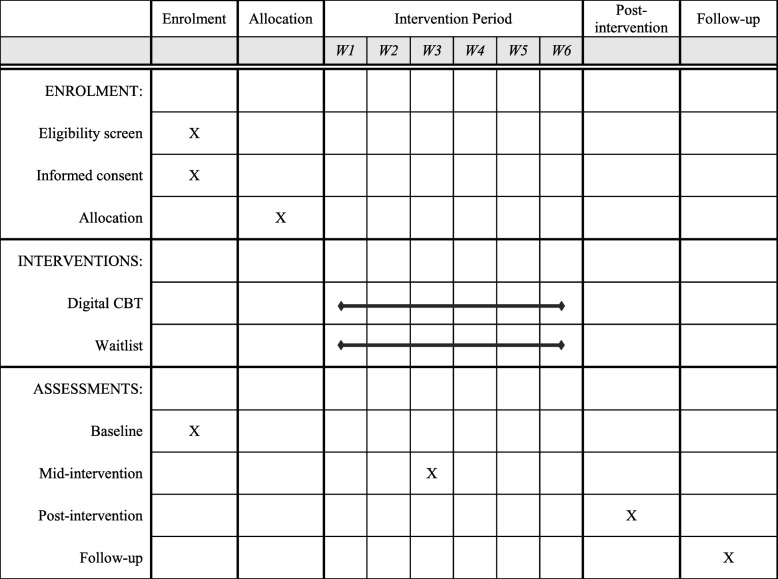
Schedule of enrolment, intervention, and assessments. CBT cognitive behavioural therapy, W week

We will publish the results of this study in peer-reviewed journals, irrespective of magnitude or direction of effect. Findings will also be presented at national or international scientific meetings. A lay report of the findings will be produced and disseminated to participants upon request. Items in this protocol comply with the Standard Protocol Items: Recommendations for Interventional Trials (SPIRIT) checklist [53] (see Additional file 1; and Fig. 2 for the SPIRIT figure).

### Ancillary and post-trial care

Any participant who is discovered to have another medical condition will be referred to their medical practitioner by a study clinical psychologist. Participants who complete or withdraw at any time for any reason during the study period will be referred by a study clinical psychologist at discretion, and/or if required or requested by the participant, to their medical practitioner for further management.

## Discussion

Despite the evidence base for CBT for GAD [16, 17] there are substantial barriers to accessing this treatment (e.g. insufficient numbers of trained therapists, costs, waiting lists, distance from services, stigma) [18, 19]. Digital CBT not only provides a solution to overcoming treatment accessibility barriers, but also has the potential to provide standardised treatment that can be personalised to the individual. This protocol is for a two-arm parallel-group superiority RCT examining the efficacy of a novel and fully automated smartphone-delivered CBT intervention compared to waitlist control for moderate-to-severe symptoms of GAD. This trial also explores worry as a potential mechanism underlying the effect of digital CBT for GAD on anxiety symptom severity.

Findings have the potential to contribute towards the evidence base for digital CBT for GAD and increasing the dissemination of CBT. Given the novelty of the smartphone digital CBT intervention for GAD currently assessed, this trial compares the intervention to a waitlist control group. Building on findings from efficacy trials of this digital CBT for GAD intervention, it would be helpful for future research to further illuminate how and for whom digital CBT for GAD yields therapeutic benefits and whether digital CBT for GAD confers specific benefits by comparing this to active control conditions. If findings support the efficacy of the intervention, it would also be valuable to examine effectiveness in larger participant samples across different settings.

### Trial status

This is protocol version 1.1, September 2019. Recruitment started on 2 August 2019.

At the time of manuscript submission, recruitment for this study is not yet complete. It is anticipated that recruitment will end in November 2019.

### SPIRIT guidelines

Please see Fig. 2 for a copy of the SPIRIT figure. The SPIRIT checklist can be found in Additional file 1.

## Supplementary information

**Additional file 1.** SPIRIT 2013 checklist: recommended items to address in a clinical trial protocol and related documents.

## Data Availability

The full study protocol, statistical code and participant-level dataset for the current study may be available from the corresponding author on reasonable request.

## Abbreviations

CBT: Cognitive behavioural therapy
CONSORT: Consolidated Standards of Reporting Trials
GAD: Generalised anxiety disorder
GAD-7: Seven-item Generalised Anxiety Disorder questionnaire
MINI: Mini-International Neuropsychiatric Interview
PHQ-9: Nine-item Patient Health Questionnaire
PSWQ: Penn State Worry Questionnaire
RCT: Randomised controlled trial
SCI-8: Eight-item Sleep Condition Indicator
SPIRIT: Standard Protocol Items: Recommendations for Interventional Trials
WEMWBS: Warwick–Edinburgh Mental Wellbeing Scale
